# Breeding Habitat Preference of the Dengue Vector Mosquitoes *Aedes aegypti* and *Aedes albopictus* from Urban, Semiurban, and Rural Areas in Kurunegala District, Sri Lanka

**DOI:** 10.1155/2024/4123543

**Published:** 2024-01-29

**Authors:** J. M. Manel K. Herath, W. A. Priyanka P. De Silva, Thilini C. Weeraratne, S. H. P. Parakrama Karunaratne

**Affiliations:** ^1^Entomological Surveillance Unit, Office of Regional Director of Health Services, Kurunegala, Sri Lanka; ^2^Postgraduate Institute of Science, University of Peradeniya, Peradeniya, Sri Lanka; ^3^Department of Zoology, Faculty of Science, University of Peradeniya, Peradeniya, Sri Lanka

## Abstract

Elimination of vector mosquito larvae and their breeding environments is an effective strategy in dengue disease control. Present study examined larval density and water quality in breeding habitats and container preference of dengue vectors *Ae. aegypti* and *Ae. albopictus*. Larval surveys were conducted monthly in urban, semiurban, and rural sites in Kurunegala, Sri Lanka, from January 2019 to December 2021. Larval densities were recorded under the following three categories: type of container (16 types), type of material (6 types), and location (indoor/outdoor). Breeding preference ratios (BPRs) were calculated using Index of Available Containers and the Index of Contribution to Breeding Sites. Out of 19,234 wet containers examined, larval stages were found in 1,043 habitats. *Ae. albopictus* larvae were in all three areas whereas *Ae. aegypti* larvae were restricted to urban areas. Highest number of wet containers and highest positivity were reported from urban followed by semiurban. In general, discarded nondegradable items were the most frequent and mostly positive breeding sites. For *Ae*. *aegypti*, the most preferred breeding sites were gutters and concrete slabs. *Ae. albopictus* mostly preferred concrete slabs in urban areas and tyres in semiurban and rural areas. Material types such as rubber and concrete were mostly preferred by *Ae. aegypti* whereas ceramic was preferred by *Ae. albopictus*. Although plastic was the most available material type in all study sites, preference to plastic was low except for urban *Ae. albopictus*. Both species preferred urban indoor breeding habitats although outdoor breeding was preferred by *Ae. albopictus* in rural areas. Larval densities of *Ae. aegypti* and semiurban *Ae. albopictus* significantly correlated with the BPR of the container type and material type. Dengue vector larvae were found in a 6.7–9.4 pH range. Total dissolved solids and alkalinity positively correlated with preference. Information generated can be successfully used in waste management and public education for effective vector control.

## 1. Introduction

Dengue is a mosquito-borne disease that has rapidly spread across the world [1]. It is caused by the DENV virus of the genus *Flavivirus*. DENV has the following four serotypes: DENV 1 to DENV 4. The disease is transmitted through the bites of infected *Aedes* mosquitoes [2]. *Ae. aegypti* Linnaeus and *Ae*. *albopictus* Skuse are considered as the most prominent dengue vector species in the world [2], and their close association to human habitations and acquired human host specificity have enabled them to display high vector competence giving high infection and transmission rates for dengue and other viruses [3]. They are considered container breeders and breed in indoor and outdoor settings in a wide variety of natural and man-made water-holding containers, such as discarded plastic containers, plant axils, water storage barrels, cement tanks, and flower pots. *Ae. aegypti,* which is considered the primary vector of dengue, is highly anthropophilic and thrives close to human inhabiting mainly in urban and periurban areas [4, 5] and commonly breeds in artificial containers in houses [6]. *Ae. albopictus*, the secondary vector of dengue, is a sylvatic species that has recently been adapted to urban and semiurban environments but still tends to breed more often in natural containers, such as tree stumps and coconut shells, and to a lesser extent in artificial containers [6].

Even though more research studies are needed to explain the factors responsible for the attraction of dengue vectors to diverse breeding sites, many researchers have identified breeding habitat availability as the key factor that determines the preference [7–10]. In most cases, data from larval surveys are used to calculate the breeding preference ratios based on positivity which may have little to do with productivity. It is important to understand the association between breeding habitat physicochemical parameters and larval productivity. Water quality parameters in breeding environments play a significant role in egg hatching and growth of the progeny from larvae to adults [11, 12]. Because of this, gravid female mosquitoes are sensitive to both biotic and abiotic factors such as organic matter [12], bacteria, phosphate, ammonia, and potassium content [13] of the water during the breeding exercise. These factors are known to be closely related to the abundance of larvae and adults in the field [14]. Although presence of heavy metals such as iron, zinc, and copper has been found at various concentrations in *Ae. aegypti* breeding sites [15, 16], their relationship with larval development remains unknown.

Type of the containers, such as buckets, bottles, vases, and tanks, has been searched by many researchers [8–10] while some others have used other criteria, such as material type and the capacity [9, 17], together with the container type for their studies. None of these studies, however, has incorporated features like attractiveness of female mosquitoes or functional characteristics as influenced by human actions to breeding sites. Lack of uniformity among container classifications has made the situation worst in comparing the results and decision-making strategies.

In order to formulate successful vector control strategies, the current study attempted to establish the association between larval density and container preference based on the container availability, container type, materials of container, location, and water quality.

## 2. Materials and Methods

### 2.1. Study Sites

**Figure 1:**
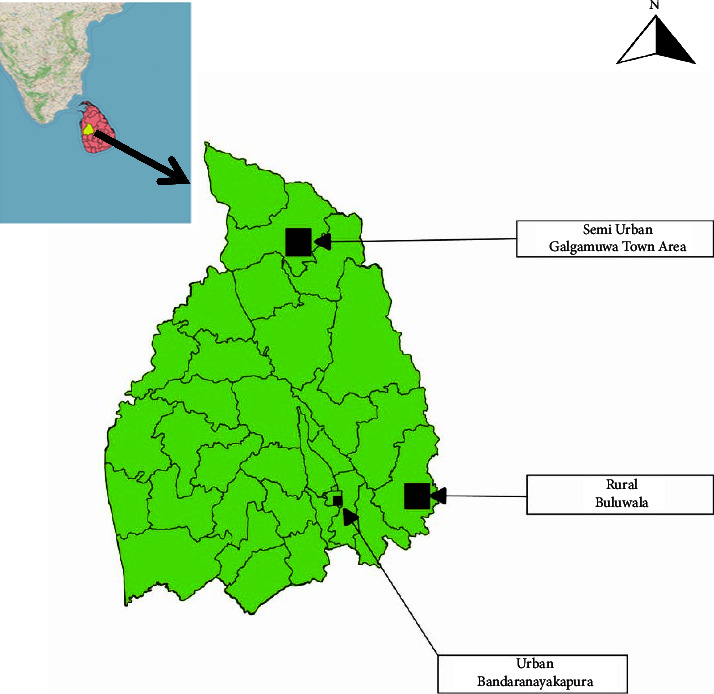
Map of the study locations in the Kurunegala district, Sri Lanka. Breeding habitats of *Ae. aegypti* and *Ae*. *albopictus* were studied in three localities (Bandarnayakapura, Galgamuwa town area, and Buluwala).

Three localities, i.e., rural Buluwala (BUL) in Rideegama Medical Officer of Health (MOH) area, semiurban Galgamuwa (GAL) in Galgamuwa MOH area, and urban Bandaranayakapura (BAN) in Kurunegala MOH area, were selected for the study from Kurunegala district (longitude: 80.1875065; latitude: 7.5869294; and elevation: 76 m), Sri Lanka (Figure 1). All three locations had been classified as dengue high-risk areas and had >500 dengue cases per year for the five-year period before the study [18] which was conducted from January 2019 to December 2021.

Bandaranayakapura (96 km^2^) with more than 35,453 population (600 buildings) including densely situated houses, stores, and offices was considered as an urban area. Galgamuwa (278.4 km^2^) with around 19,987 population (500 buildings), and with somewhat lower sanitary facilities and no municipal water supply, was considered as a semiurban area. Buluwala (220 km^2^) with nearly 9,789 population (400 buildings) living under poorer economic conditions, encircled by mountains and thick vegetation, was considered as a rural area. The endemicity of the disease during the study period was 622 cases in 2019 (Kurunegala: 278, Galgamuwa: 189, and Rideegama: 155), 317 cases in 2020 (Kurunegala: 164, Galgamuwa: 62, and Rideegama: 91), and 692 cases in 2021 (Kurunegala: 378, Galgamuwa: 162, and Rideegama: 152).

### 2.2. Larval Survey

Only the sites maintained by home owners (residential areas) were investigated. Mosquito larval surveys were conducted at all selected sites following the systematic sampling strategy [19]. Every second premise along the locality's inspection route was chosen for sampling. A total of 108 surveys were completed over the study period from all study areas. For one survey, a minimum of 100 locations per site were selected for sampling. At each location, all water-holding containers, in both indoors and outdoors, were inspected for the presence of larvae. A container with at least one immature mosquito was considered as a positive breeding site. Dippers were used to sample 50 mL from larger water-holding bodies, and depending on the water volume, up to three samples were taken from varying depths. Larval density per container was calculated as the number of larvae per 50 mL volume. Surveys were conducted monthly during January 2019 to December 2021 period in all three study sites.

Collected larvae were brought to the water laboratory at the office of the regional director of health services, Kurunegala, fixed in 70% ethanol and identified using the key introduced by Darsie and Ward [20]. Water samples were collected separately from each type of breeding habitats for quality analysis.

### 2.3. Characterisation of Dengue Vector Breeding Habitats

**Figure 2:**
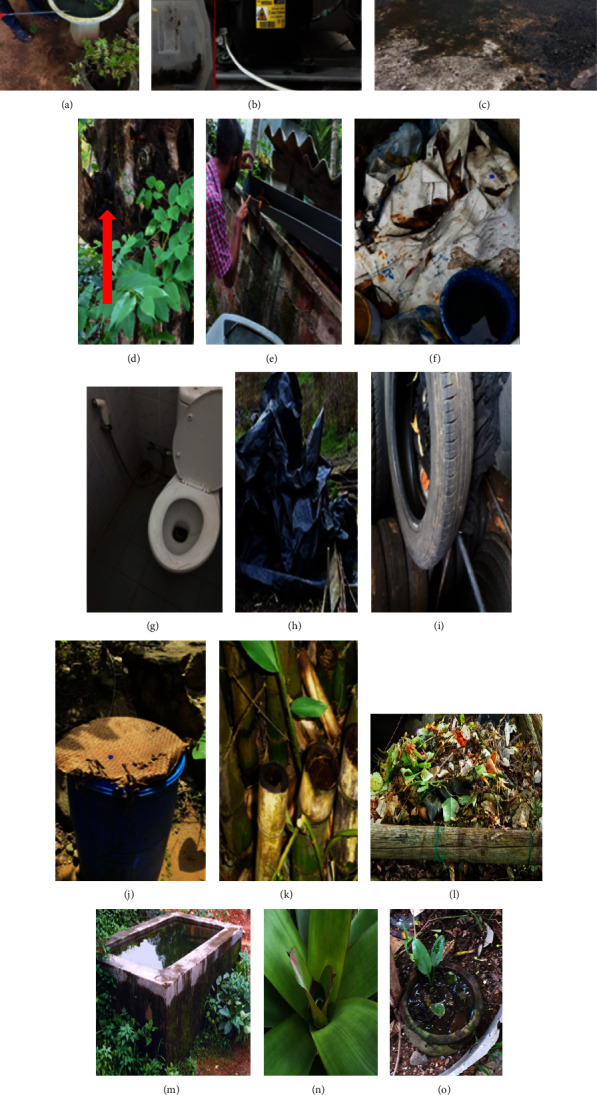
Different types of breeding habitats. (a) Ornamental flower pot, (b) fridge tray, (c) concrete slab, (d) tree hole, (e) gutter, (f) discarded nondegradable receptacles, (g) commodes and cisterns, (h) covering items, (i) tyres, (j) water storage barrel, (k) bamboo stumps, (l) discarded degradable receptacles, (m) water storage cement tanks, (n) leaf axis, and (o) clay pots.

Water-holding positive and potential containers were classified based on their usage type, material, and placement. The usage type was classified into sixteen categories considering the use indicated by the homeowners, i.e., concrete slabs, gutters, water storage barrels water storage cement tanks, tyres, ornamental flower pots, tree holes, leaf axes, bamboo stumps, and clay plots, AC refrigerators, used/nonused commodes and cisterns, discarded degradable items (coconut shells, decaying leaves, kitchen waste, paper waste, damaged paper boxes, etc.), discarded nondegradable items (tins, yoghurt and ice cream cups, bottles, cans, damaged ceramic items, etc.), and covering items. All other breeding habitats were classified as miscellaneous (Figure 2).

The materials of the containers were classified into eight categories, i.e., concrete, plastic, natural, clay, ceramic, rubber, glass, and miscellaneous (tin, paper-based material, and so on). Based on the placement, containers were classified as indoor and outdoor.

### 2.4. Measurement of Water Quality Parameters

Water samples for analysis were collected according to the container type and the material of the container. Analysis required about 1 L volume from each container type, and for the containers with smaller capacities, water from the same container type was combined to make 1 L volume. Water in the containers coming under miscellaneous category, leaf axis, and tree holes were not analysed due to the low water volumes obtained. Minimum of 3 one liter samples from each container type were analysed.

Six water quality parameters were tested; that is, pH readings were measured with a pH meter (Eutech Instrument, PC 700), free ammonia concentration was measured using Lovibone Nessterizer with Lovibond® water testing, total alkalinity and chloride concentration were determined using titrimetric methods according to the standard procedures given by the Sri Lanka Standard Institution [21], TDS content was measured using TDS meter (Bench type conductivity, Eutech Instrument), and total iron concentration was determined using a spectrophotometer (UV/VIS Spectrodirect-German).

### 2.5. Data Analysis

For each breeding habitat, three indices were calculated based on the three container grouping categories. The Index of Aailable Containers (IACs) was calculated as the total number of containers of a particular type from each category divided by the total number of containers in the residences. The Index of Contribution to Breeding Sites (ICBSs) was calculated as the number of positive containers from each category divided by the total number of positive containers in the residences. The breeding preference ratio (BPR) was calculated as the ratio of ICBS to IAC for each category [22]. A value less than one indicates that the category is not attractive to female mosquitos, whereas values more than one indicate that the category is exploited. A value of one would suggest that the particular container category is used in the same proportion as it is available [22].

The Pearson correlation and linear regression analysis was utilized to determine the container variables related to the abundance of *Ae. aegypti* and *Ae. albopictus* larvae. One-way ANOVA and Tukey's pairwise comparison tests were used to make comparisons. Variation of each of the physicochemical characteristics between breeding sites was determined by one-way ANOVA. Pearson's correlation coefficient (*r*) analysis was used to explore the association between physicochemical parameters and mosquito breeding preference. Multivariate studies, which included principal component analysis (PCA) and cluster analysis, were carried out using XLSTAT Version 2012.2.03 to investigate the link between each variable and the group based on similarity levels.

## 3. Results

### 3.1. Prevalence of Containers and Their Positivity for Dengue Vector Larvae

During the period of January 2019 to December 2021, 14,452 premises were investigated from all three study sites. Highest number of water-holding containers (wet containers) were reported from the urban area (*n* = 10,578; 54.73%) followed by the semiurban (*n* = 4,424; 23.07%) and rural (*n* = 4,232; 22.2%) areas. Dengue vector larval stages were found in 1043 (5.4%) of the water-holding containers. *Ae. aegypti* was reported only from the urban area, whereas *Ae. albopictus* was found from urban, semiurban, and rural areas. Highest positivity was reported from the urban area (*n* = 603; *Ae. aegypti* only = 114; *Ae. albopictus* only = 477; both species = 12), followed by semiurban areas (*n* = 247; *Ae. albopictus* only) and rural areas (*n* = 193; *Ae. albopictus* only). Overall mixed breeding percentage was 0.03%.

#### 3.1.1. Prevalence according to the Type of Container

**Figure 3:**
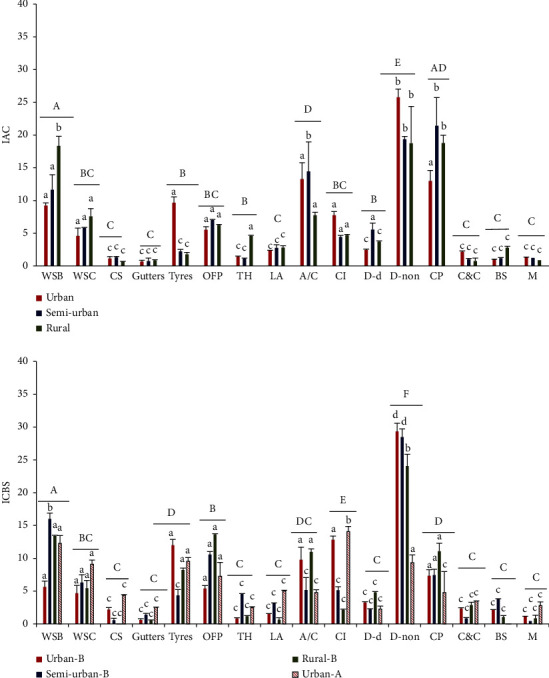
Container availability (given by Index of Available Containers (IACs)) and their positivity (given by Index of Contribution to Breeding Sites (ICBSs)) for dengue vector species (A: *Ae. aegypti*; B: *Ae. albopictus*) for the urban, semiurban, and rural study areas. Different letters indicate significant differences (*p* < 0.05) among the habitats (a–d) and among the groups (A–E lines above the groups) by analysis of variance (ANOVA). WSBs: water storage barrels, WSC tanks: water storage cement tanks, CSs: concrete slabs, OFPs: ornamental flower pots, THs: tree holes, leaf axis: LA, AC: air conditioning refrigerators, CIs: covering items, D-d: discarded degradable, D-non: discarded nondegradable, CPs: clay pots, C and C: commodes and cisterns, BSs: bamboo stumps, and M: miscellaneous.

Index of Available Containers (IACs) for different container types is shown in Figure 3. On average, highest IAC was reported for discarded nondegradable items (*n* = 4423, 23.01%) followed by clay pots (*n* = 3269, 16.91%). In urban areas, discarded nondegradable items had the highest IAC, which was significantly higher (df = 14, *F* = 11.23, *p* = 0.002) than that reported for other two areas (Figure 3). In semiurban areas, highest IAC was for clay pots and it was significantly higher (df = 14, *F* = 13.23, *p* = 0.02) than that for other areas. In rural areas, water storage barrels had the highest IAC which was significantly higher (df = 14, *F* = 14.67, *p* = 0.003) than that reported for other two areas (Figure 3). Larval density (larvae per 50 ml volume of water) of all container types significantly correlated with their respective IAC values, and the association was stronger for urban *Ae. albopictus* (*p* = 0.001, *r* = 0.78). Covering items (14.23 ± 3.3) and water storage barrels (12.41 ± 2.5) had the highest ICBS values for urban *Ae. aegypti* (df = 14, *F* = 13.71, *p* = 0.008). The highest ICBS value for *Ae. albopictus* was reported for discarded nondegradable items (urban 29.32 ± 1.57, semiurban 28.5 ± 5.7, rural 24 ± 3.5) (Figure 3). For both vector species, larval density of all container types significantly correlated (*p* < 0.05) with their respective ICBS values (for urban*Ae. aegypti*, *r* = 0.831; for urban*Ae. albopictus*, *r* = 0.933; for semiurban, *r* = 0.922; and for rural, *r* = 0.874).

#### 3.1.2. Prevalence according to the Type of Material

**Figure 4:**
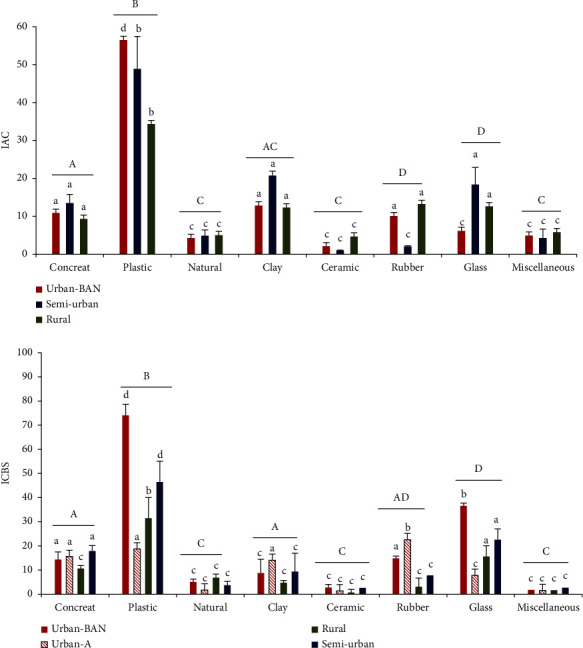
Container availability (IAC) according to the material type and their positivity (ICBS) for dengue vector species (A: *Ae. aegypti*; B: *Ae. albopictus*) in the urban, semiurban, and rural study sites in Kurunegala district. Different letters indicate significant differences (*p* < 0.05) among the habitats (a–d) and among the groups (A–D lines above the groups) by analysis of variance (ANOVA).

Plastic was the most available material type of wet containers in all three areas (Figure 4) as shown by highest IAC values (urban = 56.5 ± 4.6, semiurban = 48.9 ± 8.56, and rural = 44.9 ± 8.6) (df = 7, *F* = 13.8, *p*=0.011). Larval density significantly correlated (*p* < 0.05) with IAC for all container material types, especially with *Ae. albopictus* for all study areas (*Ae. albopictus r* = 0.70; *Ae. aegypti r* = 0.478).

The ICBS values were significantly different among different breeding habitat material types for both *Ae. aegypti* (df = 7, *F* = 32.67, *p*=0.023) and *Ae. albopictus* (df = 7, *F* = 33.1, *p*=0.001). Rubber had the significantly highest ICBS value (mean = 22.6 ± 3.1) for *Ae. aegypti* and plastic materials (urban: 74.1 ± 7.6; semiurban: 46.5 ± 3.9; rural = 31.4 ± 5) had significantly highest ICBS value for *Ae. albopictus* (Figure 4). Larval density significantly correlated with ICBS for all material types for all three study area *Ae. albopictus* (*p*=0.001, *r* = 0.90) but not for urban *Ae. aegypti*.

#### 3.1.3. Prevalence according to the Place of Container

**Figure 5:**
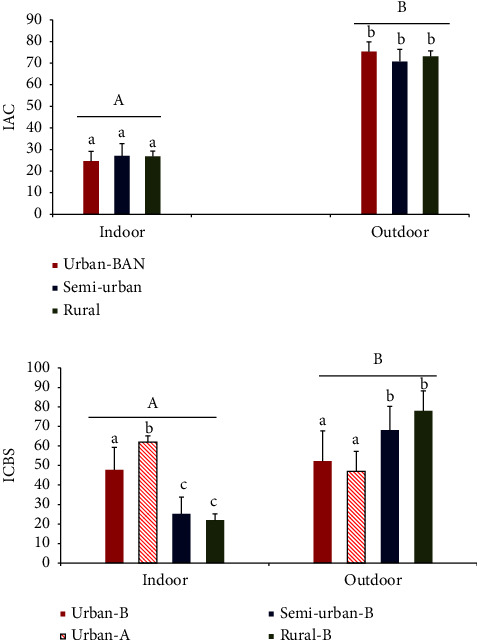
Container availability (IAC) according to place of containers and their positivity (ICBS) for dengue vector species (A: *Ae. aegypti*; B: *Ae. albopictus*) for the selected urban, semiurban, and rural areas in Kurunegala district. Different letters indicate significant differences (*p* < 0.05) among the habitats (a–c) and among the groups (A-B lines above the groups) by analysis of variance (ANOVA).

Indoor IAC was significantly lower than the outdoor IAC (df = 1, *F* = 12.3. *p*=0.001) (Figure 5) for each area. No significance difference was observed among the indoor IACs and among the outdoor IACs in all three areas. *Ae. albopictus* larval density significantly correlated (*p* < 0.05) with respective indoor IAC values in semiurban and rural areas (*r* > 0.9).

Indoor ICBS value was significantly higher than the outdoor ICBS value for *Ae. aegypti* (df = 1, *F* = 10.23, *p*=0.002) in urban areas (Figure 5). No significant difference was observed between indoor and outdoor ICBS values for *Ae. albopictus* in urban areas. Indoor ICBS values were significantly lower than the outdoor ICBS values for *Ae. albopictus* in semiurban (df = 1, *F* = 11.98, *p*=0.02) and rural areas (df = 1, *F* = 10.54, *p*=0.001).

### 3.2. Breeding Preference Ratio for Dengue Vectors in Urban, Semiurban, and Rural Areas

For *Ae. aegypti*, the most preferred breeding container types were gutters (BPR = 3.89) and concrete slabs (BPR = 3.86). For *Ae. albopictus,* the most preferred were concrete slabs (BPR = 1.98) in urban areas and tyres in semiurban (BPR = 4.56) and rural (BPR = 1.66) areas. Tyres and discarded nondegradable items had >1 BPR value in all study sites for *Ae. albopictus* (Table 1).

With regard to the material types of the containers, rubber (BPR = 2.25) followed by concrete (BPR = 1.43) had high BPR value for *Ae. aegypti*. For *Ae. albopictus*, ceramic (urban = 1.34; semiurban = 2.40; rural = 1.04) and rubber (urban = 1.48; semiurban = 3.64; rural = 1.97) had >1 BPR values in all study sites. Although the plastic material type was the most available in all study sites, plastic had <1 BPR in all study areas except for urban *Ae. albopictus* (Table 1). Breeding preference ratio (BPR) was >1 only for urban indoor breeding habitats. In rural areas, outdoor breeding habitats had >1 BPR for *Ae. albopictus* (Table 1).

A multiple linear regression analysis revealed that *Ae. aegypti* larval density significantly correlates (*p* < 0.05) with the BPR of the type and material of containers, i.e., WSB (*r* = 0.978), WSC (*r* = 0.756), gutters (*r* = 0.748), tyres (*r* = 0.999), OFP (*r* = 0.542), AC refrigerators (*r* = 0.978), covering items (*r* = 0.304), discarded nondegradable (*r* = 0.267), clay pots (*r* = 0.979), plastic (*r* = 0.994), rubber (*r* = 0.996), clay (*r* = 0.979), and concrete (*r* = 0.978), but not with the place of container. For *Ae. albopictus*, a strong association was observed between larval density and BPR based on the type of containers (*R*^2^ = 52.89, *p* < 0.05) and the material of containers (*R*^2^ = 67.45, *p* < 0.05) in semiurban areas (Table 1).

### 3.3. Water Quality Parameters

Water quality analysis results showed that TDS content of the breeding sites ranged from 60 (±12.7) mg/L in commodes and cisterns to 1547 ± 374 mg/L in tyres. Free ammonia level ranged from 0.08 (±0.001) mg/L in bamboo stumps to 0.271 (±0.24) mg/L in discarded degradable habitats. Total alkalinity range was 38 (±13.9) mg/L in commodes and cisterns to 182.8 (±36.2) mg/L in tyres. Total iron ranged from 0.04 (±0.02) mg/L in clay pots to 0.56 (±0.14) in gutters. The highest chloride concentration was from AC refrigerators (632 ± 528 mg/L) whereas bamboo stumps, covering items, discarded items, gutters, and slabs reported zero chloride concentrations. Dengue vector larvae were found in a wide pH range (6.7 ± 0.23 to 9.4 ± 0.5) (Table 2).

According to material type of containers, highest TDS, pH, free ammonia, and total alkalinity were from rubber. Highest iron content was from concrete breeding habitats. Highest chloride content was from plastic and rubber (Table 3).

**Figure 6:**
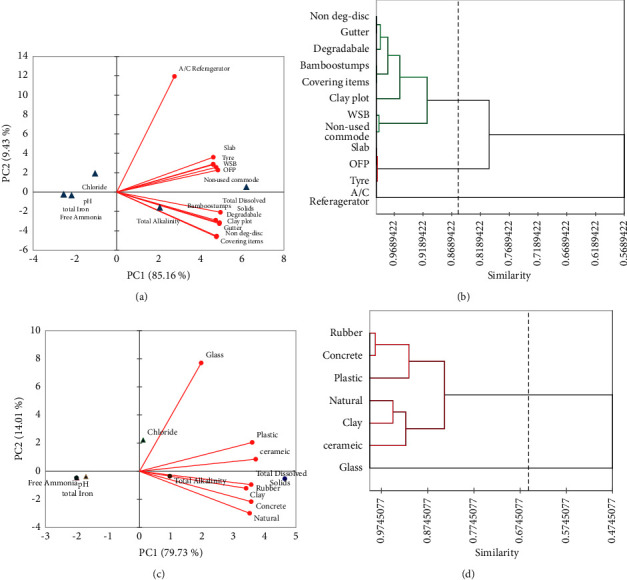
Principal components analysis (PCA) and agglomerative hierarchical clustering/dendrogram. (a, c) Correlation between water quality parameters and various container types and materials. (b, d) Classification of distinct container based on agglomerative hierarchical clustering (AHC) methodologies (clustering dendrogram) on the container type and the material of the containers.

Principal component analysis (PCA), using the physicochemical parameters of different breeding habitats according to the type and the material of containers, was carried out. A biplot analysis was used to visualize water variable correlations with container categorization (Figure 6). Results showed that water storage barrels, slabs, ornamental flower plots, tyres, and nonused commodes significantly and positively correlate with TDS. Gutters, bamboo stumps, covering items, discarded nondegradable, discarded- degradable items, and clay plots significantly associated with both TDS and alkalinity. AC refrigerators significantly associated with chloride concentration (Figure 6(a)). Three major clusters of containers were obtained based on the agglomerative hierarchical clustering (AHC) technique (Figure 6(b)).

All the container types, except AC refrigerators, and all the material types, except glass, significantly and positively correlated with TDS and alkalinity of water (Figures 6(a) and 6(c)). Two major clusters were obtained based on the similarity of water quality of both the container type and based on the similarity of water quality of the container material (Figures 6(b) and 6(d)). AC refrigerators (container type) and glass (container material) gave smaller clusters with isolation showing their distinctive water quality profiles compared to others. According to the Pearson correlation coefficient analysis carried out, *Aedes* larval density significantly associates (*p* < 0.05) with TDS (*r* = 0.31), alkalinity (*r* = 0.38), and total iron concentration (*r* = 0.16).

## 4. Discussion

Dengue fever (DF) has emerged as a serious public health concern in Sri Lanka, with an alarming increase in the number of reported cases. Development of resistance against synthetic insecticides has become a serious global issue threating insecticide-based vector control programmes [23, 24]. Considering the risk of resistance, cost effectiveness, environmental acceptance, and long-term influence, dengue vector control efforts in Sri Lanka are primarily focused on larval source reduction [25–27]. Therefore, establishing an extensive knowledge on breeding environments and vector preference for breeding places has become crucial. In Sri Lanka, several research studies have been carried out on the availability and positivity of *Ae*. *aegypti* and *Ae*. *albopictus* breeding habitats and their characteristics [25, 28–32]. The current study focused on different aspects of mosquito breeding habitats including habitat preference, chemical nature of the breeding water, and their association with larval density which are important for risk assessment and to develop effective vector control strategies [2]. The study design was characterized by its diverse breeding habitat exploration and attention to sample size in order to provide a comprehensive understanding of dengue mosquito breeding preferences.

Our survey results from rural, semiurban, and urban environments revealed that *Ae. albopictus* is the predominant species in all three areas and *Ae. aegypti* is restricted to urban areas. Prevalence of the arboviral vector *Ae*. *aegypti* in urban areas has been attributed to its dependence on human dwellings for blood meals [33, 34]. Higa et al. [35] demonstrated that *Ae. albopictus* was more associated with natural habitats (e.g., tree holes, bamboo stumps, and bromeliads) and considered it a rural vector. Our results also showed that *Ae. albopictus* prefers outdoor breeding. However, this species has now become adapted to urban environment also, breeding in artificial containers to become the predominant vector in urban environment [36–38]. Replacement of *Ae. aegypti* with *Ae. albopictus* in urban environments has been observed in many countries throughout the world [36–39]. Most probable reason for this replacement is the successful competitiveness of *Ae. albopictus* over *Ae. aegypti* for breeding habitats [40, 41].

The present study demonstrated that discarded nondegradable items were the most frequent and mostly positive breeding sites in all the study areas, confirming previous reports available in the literature [10, 42]. Larval productivity increases with the availability of the waste containers, and thus appropriate measures should be taken for waste management. Asian productivity organization [43] explained that waste generation is linked with socioeconomic factors, which are expected to differ between urban and rural communities. Although life style patterns differ between urban and rural areas, it appears that the generation of waste amount in rural areas differs quantitatively but not qualitatively from that in urban areas. Therefore, positivity varied across the three study sites, with discarded nondegradable objects being more positive in the urban than the other two. Waste collection is considerably lower in rural regions than in urban where rapid population expansion, industrialisation, urbanization, and increased consumptions take place. Dharmasiri and Dharmasiri [44] identified several challenges, including inefficient waste segregation, poor waste collection mechanisms, and lack of public commitment on waste management in urban areas. Thus, the prevailing system of waste collection, transportation, and disposal is believed to be an issue that needs to be resolved. In this context, awareness through education and changing the attitudes of the public can help to establish proper and sustainable waste management practices. Current study results highlight the abundance of discarded clay pots in semiurban areas. Many of these pots are used to pack curd commercially, and once the curd is consumed, pots are discarded to the environment by consumers. These empty clay pots are used for variety of purposes, including providing drinking water for pet animals, and subsequently turn in to mosquito breeding grounds. Water storage barrels were identified as major mosquito breeding habitats in rural areas. Lack of an adequate pipe borne water delivery system has compelled the people live in rural areas to store water in these barrels. Public awareness and education about vector breeding environment and proper water delivery system to rural areas will reduce vector breeding incidence. Plastic was identified as the material which largely contributes for diverse types of breeding habitats. Legislations should be introduced to minimise the use of plastic materials as a part of vector control strategies. Although rubber did not show a significantly higher prevalence, it was the most preferred breeding material as it contributes to tyres. Low prevalence could positively associate with breeding preference if the breeding habitat contributes to a high larval density [12, 45].

Although previous studies had specified that TDS levels of *Aedes* breeding habitats are low [27, 46–48], our results revealed that dengue vectors can survive in a broad range of TDS and the level of TDS positively correlates with BPR of *Ae. aegypti* and *Ae. albopictus*. Although the larvae were found in a wide range of pH, it has been reported that sites with high pH due to free ammonia are not ideal for mosquito breeding and survival, and a neutral pH range from 6.8 to 7.2 at breeding sites is preferred by mosquitoes [49]. Surviving in a wide range of TDS levels, pH and chloride concentrations, may reflect the adaptive nature of two vector species. Brackish water tolerance of both *Ae. aegypti* and *Ae. albopictus* has also been reported previously [50, 51]. Wang et al. [52] suggested that human interventions such as organic and nutrient pollution make a major impact on the water quality of mosquito breeding sites. However, differences in water quality could also be linked to the nature of the usage of the container, natural, artificial, or material source [53, 54].

The current investigation demonstrated the most available container types and their relationship with water quality metrics, and vector breeding preference in urban, semiurban, and rural settings in a district where dengue incidence is high. Our data provide valuable information to formulate proper waste management plans, public education, and awareness programmes for an effective vector control.

## 5. Conclusions

*Ae. albopictus* is the predominant vector found in all three urban, semiurban, and rural areas while *Ae. aegypti* is limited to urban areas. Discarded nondegradable items were the most prevalent container type, and plastics were the prominent material type in all study sites. Although the prevalence was low, tyres were the highest preferred breeding site for both species. Both vectors were present in a wide variety of water quality conditions showing their high adaptability. Information gathered can be used to formulate successful waste management plans, public education programmes, and effective vector control practices.

## Figures and Tables

**Table 1:** Correlation between larval densities of dengue vectors (A: *Ae. aegypti* and B: *Ae. albopictus*) and breeding preference ratio (BPR) according to container categories in urban, semiurban, and rural study sites of Kurunegala district.

Container category		*BPR*				*Larval density*				*Correlation between BPR and larval density*			
		Urban-B	Semiurban-B	Rural-B	Urban-A	Urban-B	Semiurban-B	Rural-B	Urban-A	Urban-B	Semiurban-B	Rural-B	Urban-A
Type of container	WSB	0.63	1.33	0.85	1.33	22.33	27.00	45.67	15.00	0.685	0.865^*∗*^	0.719	0.978^*∗*^*∗*^^
	WSC	0.99	0.91	0.92	1.98	20.33	21.33	28.67	14.00	0.976	0.999^*∗*^	0.976^*∗*^	0.756^*∗*^
	Concrete slabs	1.98	0.00	1.12	3.86	16.33	9.67	5.00	11.00	0.276	0.967^*∗*^	0.453	0.785
	Gutters	0.44	0.31	1.25	3.89	4.67	1.33	12.67	21.00	0.998^∗^	0.786^*∗*^	0.345	0.748^*∗*^
	Tyres	1.40	4.56	1.66	0.99	43.00	22.67	33.00	6.00	0.264	0.836^*∗*^	0.909^*∗*^	0.999^*∗*^
	Ornamental flower pots	0.97	2.10	1.60	1.31	19.33	56.00	40.00	4.00	0.686	0.979^*∗*^	0.185	0.542^*∗*^
	Tree holes	0.64	0.97	1.00	1.79	2.67	17.33	7.00	2.00	0.263	0.331	0.559	0.178
	Leafe axis	0.85	0.10	0.83	2.11	5.00	0.67	8.67	4.00	0.971	0.672	0.706	0.542
	AC refrigerator	0.91	0.68	0.73	0.36	32.00	19.00	12.00	1.50	0.999^*∗*^	0.998^*∗*^	0.714^*∗*^	0.978^*∗*^
	Covering item	1.55	0.78	1.25	1.82	43.67	5.00	15.33	12.00	0.888	0.246	0.904^*∗*^	0.304^*∗*^
	Discarded degradable	1.32	0.96	0.61	0.92	16.33	5.00	8.00	1.50	0.716	0.863^*∗*^	0.876	0.172
	Discarded nondegradable	1.18	1.27	1.53	0.36	101.0	50.33	137.0	4.00	0.342	0.975^*∗*^	0.708	0.267^*∗*^
	Clay pots	0.55	0.50	0.40	0.37	23.00	39.00	22.67	2.00	0.867^*∗*^	0.526^*∗*^	0.646^*∗*^	0.979^*∗*^
	Commodes and cisterns	0.45	0.83	0.31	0.97	6.00	15.00	9.00	12.67	0.615	0.650	0.654	0.872^*∗*^
	Bamboo stumps	1.03	0.45	1.20	0.00	9.33	2.33	16.33	0.00	0.134^*∗*^	0.435^*∗*^	0.231^*∗*^	—
	Miscellaneous	0.21	0.12	0.11	0.23	2.76	2.45	1.65	1.23	0.143	0.254	0.345	0.123

Type of materials	Concrete	1.32	1.32	0.82	1.43	56.00	68.00	73.67	29.00	0.964	0.992^*∗*^	0.777^*∗*^	0.978^*∗*^
	Plastic	1.31	0.95	0.70	0.33	137.0	139.6	222.6	53.50	0.856	0.999^*∗*^	0.980^*∗*^	0.994^*∗*^
	Natural	1.20	0.78	1.09	0.41	17.00	6.00	32.00	6.00	0.868	0.994^*∗*^	0.948^*∗*^	0.786
	Clay	0.69	0.45	0.28	1.10	23.00	16.00	22.67	2.00	0.999^*∗*^	0.999^*∗*^	0.84	0.979^*∗*^
	Ceramic	1.34	2.40	1.04	0.68	6.00	11.33	9.00	0.00	0.191	—	—	—
	Rubber	1.48	3.64	1.97	2.25	43.00	39.67	33.00	6.00	0.696	0.854^*∗*^	0.406^*∗*^	0.996^*∗*^
	Glass	1.40	1.23	0.93	0.30	101.0	69.67	137.0	4.00	0.165	0.456^*∗*^	0.432	0.234^*∗*^
	Miscellaneous	0.22	0.24	0.26	0.31	4.62	3.87	5.87	1.67	0.132	0.243	0.145	0.456

Place of container	Indoor	1.94	0.93	0.82	1.94	60.33	75.67	66.67	3.90	0.345	0.586	0.645	0.245
	Outdoor	0.69	0.96	1.07	0.83	304.6	229.0	549.0	4.73	0.786	0.569	0.289	0.567

^*∗*^Significant correlation (*p* < 0.05).

**Table 2:** Water quality parameters of dengue vector mosquito breeding container types (mean ± SE).

Container type	Total dissolved solid (mg/L)	Free ammonia (mg/L)	Total alkalinity (mg/L)	Total iron (mg/L)	Chloride (mg/L)	pH
AC refrigerators	522 (±278)^bc^	0.082 (±0.02)^b^	100.7 (±30.5)^a^	0.29 (±0.1)^b^	632 (±528)^a^	6.7 (±0.23)^c^
Bamboo stump	98 (±3)^bc^	0.08 (±0.001)^b^	79.5 (±7.5)^a^	0	0	9.4 (±0.5)^a^
Clay plot	124.2 (±14.1)^c^	0.076 (±0.03)^b^	116.8 (±17.2)^a^	0.04 (±0.02)^b^	30.6 (±19.4)^b^	7.78 (±0.52)^ab^
Covering items	115.7 (±32.2)^bc^	0.28 (±0.26)^b^	96.7 (±57.8)^a^	0.4 (±0.1)^b^	0	7.06 (±0.2)^c^
Discarded degradable	168.7 (±5.91)^bc^	0.271 (±0.24)^b^	89 (±25.9)^a^	0.12 (±0.01)^b^	0	7.02 (±0.4)^c^
Discarded nondegradable	186 (±12.3)^bc^	0.152 (±0.04)^b^	119 (±6.76)^a^	0.13 (±0.02)^b^	0	7.47 (±0.3)^ab^
Gutters	226.8 (±12.2)^bc^	1.64 (±0.35)^a^	150.4 (±24.2)^a^	0.56 (±0.14)^b^	0	7 (±1.64)^c^
Commodes and cisterns	60 (±12.7)^c^	0.59 (±0.47)^b^	38 (±13.9)^a^	0.075 (±0.02)^b^	27.5 (±4.79)^b^	7.35 (±0.17)^b^
Ornamental flower pots	1172 (±423)^ab^	0.1 (±0.01)^b^	136.4 (±39.4)^a^	0.12 (±0.07)^b^	60 (±8.22)^b^	7.94 (±0.41)^abc^
Slab	166 (±6.22)^c^	0.16 (±0.01)^b^	98.14 (±9.98)^a^	1.37 (±0.24)^a^	0	6.98 (±0.13)^c^
Tyres	1547 (±374)^a^	0.084 (±0.004)^b^	182.8 (±36.2)^a^	0.1 (±0.04)^b^	171 (±50.2)^a^	8.82 (±0.03)^ab^
Water storage barrels	189.9 (±35.8)^c^	0.08 (±0.02)^b^	126.5 (±43.4)^a^	0.15 (±0.12)^b^	95.5 (±15.4)^b^	7.86 (±0.26)^abc^

Different letters in the same column indicate significant differences according to the Kruskal–Wallis *H* test.

**Table 3:** Water quality parameters of dengue vector mosquito breeding containers according to the material type of containers (mean ± SE).

Material type of containers	Total dissolved solids (mg/L)	Free ammonia (mg/L)	Total alkalinity (mg/L)	Total iron (mg/L)	Chloride (mg/L)	pH
Plastic	515.2 ± 124.1^a^	0.474 ± 0.07^a^	123.4 ± 34.7^a^	0.3 ± 0.001^a^	259.3 ± 167^a^	7.24 ± 0.32^a^
Concrete	585 ± 145^a^	0.14 ± 0.03^a^	114 ± 34.9^a^	0.85 ± 0.02^a^	25 ± 12.09^b^	7.8 ± 0.07^a^
Rubber	1547 ± 10.7^b^	0.8 ± 0.05^a^	184 ± 12.8^a^	0	247 ± 115^a^	8.9 ± 0.23^b^
Clay	124.2 ± 44.9^c^	0.07 ± 0.04^a^	116.8 ± 12^a^	0.04 ± 0.001^a^	30.6 ± 13.9^b^	7.78 ± 0.07^a^
Natural	161 ± 123.8^c^	0.18 ± 0.01^a^	99.1 ± 27^a^	0.131 ± 0.01^a^	0	8.28 ± 0.8^b^
Ceramic	60 ± 35.7^d^	0.59 ± 0.07^a^	38 ± 23^b^	0.075 ± 0.001^a^	27.5 ± 0.05^b^	7.35 ± 0.02^a^
Glass	78 ± 12.98^d^	0.012 ± 0.002^a^	52 ± 12^b^	0.23 ± 0.04^a^	158 ± 97.7^a^	7.45 ± 0.01^a^

Different letters in the same column indicate significant differences between different breeding habitats in each water quality parameter according to the Kruskal–Wallis *H* test.

## Data Availability

The data used to support the findings of this study are available from the corresponding author upon request.
